# Acute Abdomen Accompanied by Torsion of a Segment of the Jejunum Due to Meckel’s Diverticulum: A Rare and Interesting Case

**DOI:** 10.7759/cureus.68496

**Published:** 2024-09-02

**Authors:** Athanasios Permekerlis, Stavritsa Taxiarchoula Varvara, Eirini Gemousakaki, Christos Tepelidis, Panagiotis Fotiadis

**Affiliations:** 1 Second Surgical Department, 424 General Military Hospital, Thessaloniki, GRC

**Keywords:** meckel's diverticulum, exploratory laparotomy, torsion of meckel's diverticulum, gastrointestinal ileus, acute surgical abdomen

## Abstract

Meckel’s diverticulum is a common congenital anomaly of the gastrointestinal tract and is often asymptomatic. This case report details the diagnosis and treatment of small bowel obstruction particularly in a segment of the jejunum in a 41-year-old woman due to Meckel’s diverticulum. The patient presented with diffuse abdominal pain and bloating. Imaging and clinical evaluation revealed signs of ileus, leading to an exploratory laparotomy. Intraoperative findings included a mass consistent with Meckel’s diverticulum causing adhesion and torsion of the jejunum. The surgical intervention involved adhesiolysis and segmental enterectomy with side-to-side anastomosis. Histopathological analysis confirmed Meckel’s diverticulum with ectopic gastric mucosa. The patient had an uneventful postoperative recovery and was discharged on the seventh postoperative day.

## Introduction

Meckel’s diverticulum is the most common congenital anomaly of the gastrointestinal tract [1], occurring in approximately 2% of the population. It is called a true diverticulum and is usually located on the antimesenteric border of the ileum, typically positioned 7-200 cm away from the ileocecal valve [2]. Males and females are equally affected among incidentally found lesions, but symptomatic Meckel’s diverticulum occurs more often in males than females, with a ratio of 2-4:1. The presence of Meckel’s diverticulum is usually asymptomatic and approximately 5-6% of patients with a Meckel’s diverticulum will develop symptoms [3]. Symptoms include bleeding, intestinal obstruction, and inflammation with or without perforation [2]. Rare complications of Meckel's diverticulum involve its protrusion and strangulation in the inguinal or femoral canal, leading to Littre's hernia and De Garengeot's hernia, respectively [4]. This case report presents the diagnosis and treatment of small bowel obstruction in a 41-year-old woman due to Meckel’s diverticulum.

## Case presentation

**Figure 1:**
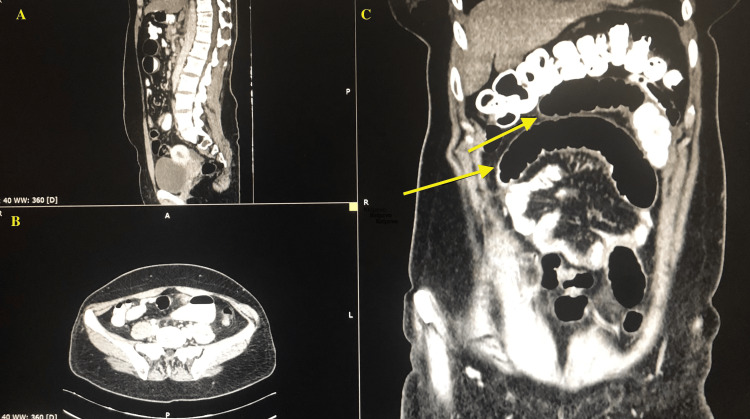
Abdominal CT scan revealing dilated small intestine loops (yellow arrows). A: Sagittal reconstruction. B: Axial reconstruction. C: Coronal reconstruction.

A 41-year-old woman presented to the emergency department of 424 General Military Hospital of Thessaloniki, complaining of diffuse abdominal pain and bloating for the past six hours. She had never experienced symptoms like this before and as for her past medical history, she had been diagnosed with polycystic ovary syndrome and irritable bowel disease and had no previous operations. The onset of symptoms followed the consumption of a large quantity of fruits. She mentioned that the pain improved with gas expulsion. The patient stated that she had two episodes of loose stools within the past 24 hours, alongside normal passing of gas. Her vital signs were within normal limits, with a blood pressure of 140/90, a pulse rate of 90 bpm, and a temperature of 36.5°C. The physical examination revealed a distended abdomen with tenderness in the lower left quadrant and hyperactive bowel sounds. Blood test results showed a slightly elevated white blood cell count of 12.73 x 10^3/μL (normal rate: 4.0-11.0 x 10^3/μL), with a predominance of polymorphonuclear cells, and negative C-reactive protein. Abdominal X-ray depicted signs of ileus with the presence of air-fluid levels and thus a CT scan was performed, without gastrografin administration in the emergency department. The CT scan revealed dilated small intestine loops with the presence of fluid-filled levels and intestinal contents without an apparent cause of obstruction, and also the presence of a pathological collection in the Douglas pouch. The patient was admitted to the surgical department for further management. Her oral intake was restricted, a nasogastric tube was inserted and intravenous fluids and IV cefoxitin were administered. Her condition remained stable and a second CT scan with the administration of gastrografin was performed two days after admission to further identify the cause of ileus (Figure 1). The CT reported dilation of the small intestine loops with mild wall thickening and a point of sharp angulation anterior to the L5 vertebra, contamination of mesenteric fat, and fluid collection in the lesser pelvis and right iliac fossa. The following day, the patient was transported to the operating room for an emergency exploratory laparotomy to further identify and manage the cause of the mechanical ileus. On exploration, a mass was found in the region of the jejunum with morphological characteristics of a Meckel’s diverticulum, surrounding this mass there were adhesions causing torsion of the segments of the jejunum. An extensive adhesiolysis and segmental enterectomy of the aforementioned segment were performed, followed by a side-to-side anastomosis with the linear cutter of 75 mm, with an isoperistaltic loop and closure of the mesentery. Additionally, an appendectomy was also performed. The postoperative period was considered uneventful and the patient was discharged on the seventh postoperative day. Histopathologic analysis of the specimen confirmed the presence of Meckel’s diverticulum with ectopic gastric mucosa.

## Discussion

Meckel’s diverticulum is a result of the incomplete atrophy of the vitelline duct (omphalomesenteric duct) during embryonic development. The omphalomesenteric duct, also known as the yolk stalk, is a structure that connects the yolk sac to the midgut of the developing embryo and is a temporary structure that obliterates between the fifth and seventh weeks of gestation [5]. The intestinal mucosa lining the walls of the ileum is also present in Meckel’s diverticulum, although it often includes ectopic tissue; the most common type of ectopic issue is gastric tissue, followed by pancreatic tissue [2]. Meckel’s diverticulum is often diagnosed as an incidental finding during surgery or an autopsy, but the appropriate method to diagnose Meckel’s diverticulum is a technetium-99m (99mTc) pertechnetate scan. It can also be diagnosed with the use of sonography, CT scan, and CT enteroclysis [6,7]. Obstruction can result from Meckel’s diverticulum acting as a lead point, causing enteroenteric or enterocolic intussusception; volvulus of the small intestine around the diverticular axis is another possible mechanism as well as entrapment of a small bowel loop around a fibrous cord or within a mesodiverticular band. Other possible mechanisms that can cause obstruction are incarceration within a hernia sac (Littre’s hernia), chronic diverticulitis, the presence of a foreign body, and the development of neoplasm [8]. In our patient, volvulus was the cause of the obstruction, which can result in serious complications if not promptly treated surgically. Notably, our patient had no history of previous surgeries, thereby ruling out adhesions as a potential cause of the obstruction. This case highlights the need for clinicians to maintain a high index of suspicion for Meckel's diverticulum in young patients who present with signs of intestinal obstruction and have no prior surgical history. Such awareness is crucial for timely diagnosis and management. As for surgical treatment, when a Meckel’s diverticulum is incidentally discovered without any symptoms, the decision for surgical resection should be based on identified risk factors and clinical judgment and it is debated. Nevertheless, symptomatic Meckel’s diverticulum requires surgical treatment. It is recommended that Meckel’s diverticula should be resected, aiming to remove the diverticulum and any associated ectopic tissue while preserving the integrity and function of the small intestine [3]. Three types of operations have been described: segmental resection-anastomosis, wedge resection, and tangential stapling [7]. In our case, laparotomy with segmental enterectomy followed by a side-to-side anastomosis was performed. The prognosis is generally good, and patient follow-up is based on evaluating clinical indications [6].

## Conclusions

Meckel’s diverticulum, though often asymptomatic, can lead to significant complications such as small bowel obstruction. Prompt diagnosis and surgical intervention are crucial for managing symptomatic cases. This case highlights the importance of considering Meckel’s diverticulum in the differential diagnosis of acute abdomen and the efficacy of surgical treatment in resolving obstruction and preventing further complications. The patient’s recovery underscores the positive prognosis following appropriate surgical management of symptomatic Meckel’s diverticulum.
